# Use of flavoured cigarettes in Poland: data from the global adult tobacco survey (2009–2010)

**DOI:** 10.1186/1471-2458-14-127

**Published:** 2014-02-06

**Authors:** Dorota Kaleta, Bukola Usidame, Anna Szosland-Fałtyn, Teresa Makowiec-Dąbrowska

**Affiliations:** 1 Department of Preventive Medicine, Medical University of Łódź, Łódź, Poland; 2 Department of Public Policy, University of Massachusetts, Boston, USA; 3 Department of Refrigeration and Food Quality in Łódź, Institute of Agricultural and Food Biotechnology, Warsaw, Poland; 4 Department of Work Physiology and Ergonomics, Nofer Institute of Occupational Medicine, Łódź, Poland

**Keywords:** Tobacco smoking, Flavoured cigarettes, Menthol cigarettes, Socio-demographic factors, Adults, GATS, Poland

## Abstract

**Background:**

Nowadays the European Union faces a debate on the ban of sale of flavoured cigarettes. There is growing evidence that certain subgroups of smokers are more vulnerable to the use of flavoured cigarettes. However in some European countries, figures on the use of these cigarettes are still scarce. The aim of the study was to assess the prevalence of flavoured cigarettes use in Poland, and examine whether its use among adults varies by socio-demographic characteristics.

**Methods:**

Data on tobacco use including flavoured cigarettes and other characteristics were derived from the Global Adult Tobacco Survey (GATS). GATS is a cross-sectional, household survey implemented in Poland between 2009 and 2010. GATS provided data on a representative sample of 7,840 individuals covering 2,254 current smokers. Logistic regression model was used to obtain odds ratios (ORs) and 95% confidence interval (CI) of the selected socio-economic variables on the use of flavoured cigarettes.

**Results:**

Among females the aromatized cigarettes use was 26.1% compared to 10.5% in males (OR = 2.3; 95% CI: 1.62–3.2; p ≤ 0.001). Respondents aged 20–29 years had an increased likelihood of using flavoured cigarettes compared to subjects aged 60 years or older (OR = 2.7; 95% CI: 1.1–6.5; p ≤ 0.001). Respondents aware of negative health consequences of smoking had OR = 1.4 95% CI: 1.1–2.1 (p ≤ 0.05) of smoking aromatized cigarettes compared to those who were unaware. Participants who perceived some kinds of cigarettes less harmful than others were also more likely to use flavoured cigarettes compared to subjects who were convinced that all cigarettes are equally harmful (OR = 1.4; 95% CI: 1.1–1.8; p ≤ 0.01). High educational attainment, living in large cities, being non-economically active was also associated with use of flavoured cigarettes.

**Conclusion:**

Our results are consistent with majority of epidemiology studies on this topic to date and should be considered in the enactment of tobacco control legislation at the national as well as European levels. For combating tobacco epidemic, further efforts need to be made to prevent smoking uptake. Ban of flavoured cigarettes could considerably support achieving this goal.

## Background

Easy access to tobacco products, low prices, advertising, promotion and sponsorship form the front line of the tobacco industry’s efforts to maintain and increase its customer base and normalize tobacco use [1]. According to the World Health Organization, one third of youth experimentation with tobacco occurs as a result of exposure to tobacco advertising, promotion and sponsorship [1]. To sell a product that kills up to half of its users requires extraordinary marketing savvy, and tobacco companies are some of the most manipulative product sellers and promoters in the world [1]. Tobacco companies have invented a number of ways to target selected groups of population through tobacco advertising despite the various bans on the use of tobacco in public places, or promoting and advertising of tobacco products. This inevitably leads to the lack of progress in tobacco smoking reduction or even an increase in smoking rates in some countries [2]. Tobacco products are carefully designed to initiate smoking and undermine efforts to quit smoking [3]. The tobacco industry introduced for example “low tar” and “light” cigarettes creating false image that those cigarettes are “softer” or even “safer” [4]. Although in many countries including Poland the usage of those terms is banned, but the tobacco industries have found a variety of ways to keep pushing their brands [1,3]. The tobacco brands use the same shape and colors which can be easily linked to the previously used terms. Furthermore, to increase tobacco consumption, the tobacco industry applies a wasteful number of ingredients to make the appearance, taste of cigarettes and smoke more attractive to the smokers, camouflage the odor of smoke released from cigarettes, and reduce its irritating effect. Cigarette content is actively manipulated by adding potentially hazardous chemical and phytochemical additives [5]. The main additions to tobacco are sugars, which are also present naturally in tobacco leaves, but moisture retaining compounds (humectants), such as glycerol and propylene glycol are added as well. They are ingredients which are part of the cigarette paper, the filter, if used, and the glue which holds a cigarette or cigar together. There are approximately six hundred additives that increase the attractiveness of tobacco products [5]. Cigarette additives have pharmacological actions that also enhance or maintain nicotine delivery, could increase the addictiveness of cigarettes, and mask symptoms and illnesses associated with smoking behaviors [5]. Examples of additives include: mitigants, glycerol, levulinic acid, sorbitol, valeric acid, isobutyl salicylate, liquorice, vanillin, methyl salicylate, menthol and many others. Finally novel, different products as well as “slim” or “flavoured” cigarettes are being introduced to attract new, younger consumers. Exotic flavours like menthol, vanilla, candy, alcohol, chocolate make the cigarettes desirable for teenagers, women and especially young ones [3,6]. It has been found that tobacco products having a characterized flavour other than tobacco may influence smoking initiation, higher exposure to smoke constituents, greater dependence on nicotine or worse smoking cessation outcomes [7-12]. Flavoured cigarettes not only have a more pleasant taste that makes smoking initiation easier, but the menthol’s cooling and anesthetic effects mask the short-term negative physiological effects of smoking such as throat pain, burning and cough. This provides superficial physical relief as well as psychological assurance against concerns about the health dangers of smoking that would otherwise motivate smokers to quit [13]. Menthol makes cigarettes easier and more palatable to smoke and less desirable to quit among established smokers. A decrease in the number of quitters contributes to the incidence of tobacco-related diseases [13] Menthol smokers, particularly women, perceive the minty aroma of menthol cigarettes to be more socially acceptable than non-flavoured cigarettes; some smokers also assume these products are healthier than regular cigarettes. However, existing evidence suggests that flavoured cigarettes are at least as harmful as the regular ones [14-16].

In December 2012, the commission came up with the proposal (Proposal for a Directive of The European Parliament And of the Council on the approximation of the laws, regulations and administrative provisions of the Member States concerning the manufacture, presentation and sale of tobacco and related products) for a revision of the European Union (EU) Tobacco Products Directive directions. EU’s Tobacco Products Directive amendments include the largest possible mandatory pictorial health warnings, standard packs, the prohibition of characterizing flavours, and the strengthening of traceability and security features for combating illicit trade. A certain concern behind the proposal is that some tobacco products like flavoured cigarettes look and taste less harmful than others, giving people a misleading impression that they are safer. The aim of the European directive is to protect young people from starting to smoke. However, proposals by the European Commission for a ban on flavoured cigarettes were not passed. Poland is one of the countries that did not support the amendments; nevertheless, decreasing the initiation rate is considered the best strategy that can determine a significant reduction in smoking-related mortality. Interventions and legislation that can decrease the smoking initiation rate on the population level could save lots of lives and money. Therefore, there is a need to increase awareness on that topic and update evidence-based data in order to work efficiently on the reduction of tobacco epidemic in Poland and other EU Member States.

The aim of the study was to assess prevalence of flavoured cigarettes use, and to examine whether the use of flavoured cigarettes among adults in Poland varies by socio-demographic characteristics. Moreover, we evaluated the differences in the awareness of the health consequences of smoking and the perception of risk of use compared to regular cigarettes from current smokers.

## Methods

We analyzed data from current male and female smokers. Data on current cigarette smoking including use of flavoured cigarettes and smokers’ socio-demographic characteristics were derived from the Global Adult Tobacco Survey (GATS) [17]. Global Adult Tobacco Survey is a cross-sectional, nationally representative household survey based on global standard protocol for systematically monitoring adult tobacco use [17]. The studied population covered non-institutional residents of Poland aged 15 years and older [18,19]. GATS was implemented in Poland between years 2008-2010. In Poland, survey population selection process was based on a three-stage stratified geographically clustered sample [17]. GATS questionnaire was very comprehensive and provided a significant number of data. The questionnaire covered detailed demands on smoking status and socio-demographic issues. Survey forms were administered at respondents’ homes during face-to-face interviews. The field work was preceded by several trainings for all survey staff and pretest. Data used for current study is publicly available from the web database of the Global Tobacco Surveillance System (GTSS). Full methodology of the GATS was described in detail elsewhere [20,21].

### Study variables

The category of the current tobacco smoking status of the respondent covered current daily and less than daily (occasionally) tobacco smokers subgroups. Current smokers were also characterized by the number of years of smoking and the number of cigarettes consumed per day. To determine the use of flavoured cigarettes, current smokers were asked questions about their last purchase of manufactured cigarettes. Questions considered the type of cigarettes that subjects have recently purchased for their own use and whether they were flavoured cigarettes, e.g. with a menthol, vanilla, or other. Data on gender and age of the respondents were also included in our analysis. We also used the data on educational attainment of all subjects in our analysis. Educational level was classified as: primary education, vocational education, secondary education, and higher education. The measure of economic activity classified subjects currently with permanent job as employed, currently with no permanent job as unemployed, and pupils, students, persons occupied with household keeping, retired, pensioners due to disability as economically non-active. Furthermore, respondents were asked whether their place of residence was a rural or urban area (urban area up to 50 000, from 50 000 to 200 000, and over 200 000 inhabitants). The total net monthly incomes of respondents were included in the analysis according to following income categories (less than 1000 PLN, from 1000 to 1500 PLN, from 1501 to 2000 PLN, from 2001 to 3000 PLN, over 3000 PLN). At the time of the study implementation, the USD exchange rate in Polish zloty currency (PLN) was 1$ USD = 3.18 PLN. We also took into consideration their awareness of the negative health consequences of smoking. We categorized our respondents as aware of negative health consequences (those who answered “yes” to the question: Do you think that tobacco smoking causes serious diseases?) and not aware (those who answered “no” and “do not know”). Moreover, people who declared to be current smokers were asked whether they perceived some kinds of cigarettes less harmful than others and two answers were considered: some kinds of cigarettes may be less harmful than others; all are equally harmful. Finally we determined the intention of current flavoured and non-flavoured users to quit. Intention to quit smoking cigarettes by respondents were recorded in four categories: intend to quit smoking within the next month; consider quitting smoking within the next 12 months; will quit smoking but not within the next 12 months; do not intend to quit smoking.

### Statistical analyses

Statistical associations of the particular categories of characteristics in the analyzed subgroups of current smokers were assessed with the chi-square test. All analyses were performed in six age groups: 15–19, 20–29, 30–39, 40–49, 50–59, 60 years and older. Logistic regression analysis was performed and the chi square test was used for trend calculation. We applied univariate and multivariate logistic regression analyses of unweighted data to obtain odds ratios (ORs) and 95% confidence interval (CI) of each indicator on the smoking of flavoured cigarettes. In the first stage crude coefficients – odds ratios (OR) of the impact of odd variables on the smoking of flavoured cigarettes in the studied group were calculated. Then, a multifactorial analysis considering the simultaneous effect of all variables on the risk of smoking flavoured cigarettes was completed. All p values were two-sided and p < 0.05 was used to indicate the statistical significance. The STATISTICA Windows XP version 8.0 program was implemented to make the statistical analysis.

## Results

Among the 14 000 households selected for the survey, 8948 (63.9%) households and 7840 (93.9%) sampled persons successfully completed the interviews. The overall survey participation rate was 65.1%. Current smokers were 2254 respondents including 1333 male and 921 female. Out of 2254 current smokers, 380 individuals declared the use of aromatized cigarettes. Among females the use of cigarettes with menthol, vanilla or other aroma was 26.1% (n = 240) compared to 10.5% (n = 140) in males (p < 0.001). More smokers, male and female, smoke regular cigarettes compared with flavoured cigarettes. The characteristics of the participants are presented in Table 1.

**Table 1:** Characteristics of users of flavoured and non-favoured cigarettes by gender – Global Adult Tobacco Survey Poland 2009–2010

**Characteristic**	**Male N = 1333**			**Female N = 921**		
	**Total smokers**	**Flavoured users**	**Non-flavoured users**	**Total smokers**	**Flavoured users**	**Non-flavoured users**
	**n**	**n (%) 140 (10.5%)**	**n (%) 1193 (89.5%)**	**n**	**n (%) 240 (26.1%)**	**n (%) 681 (73.9%)**
**Age (years)**						
15–19	38	1 (2.6%)	37 (97.4%)	19	6 (31.6%)	13 (68.4%)
20–29	252	35 (14.9%)	217 (86.1%)	163	50 (32.8%)	113 (69.3%)
30–39	305	36 (11.8%)	269 (88.2%)	183	60 (32.8%)	123 (67.2%)
40–49	304	32 (10.5%)	272 (89.5%)	223	57 (25.6%)	166 (74.4%)
50–59	267	29 (10.9%)	238 (89.1%)	241	53 (22.0%)	188 (78.0%)
> 60	167	7 (4.2%)	160 (95.8%)	92	14 (15.2%)	78 (84.8%)
p for trend		p < 0.04			p < 0.001	
**Number of years of smoking (years)**						
<1	4	0 (0.0%)	4 (100.0%)	3	0 (0.0%)	3 (100.0%)
1-2	29	1 (3.4%)	28 (96.6%)	11	2 (18.2%)	9 (81.8%)
3-4	45	2 (4.4%)	43 (95.6%)	29	8 (27.6%)	21 (72.4%)
5-9	144	21 (14.6%)	123 (85.4%)	104	33 (31.7%)	71 (68.3%)
≥10	1111	116 (10.4%)	995 (89.6%)	775	196 (25.3%)	579 (74.7%)
p for trend		p > 0.05			p > 0.05	
**Daily cigarettes consumption (cigarettes per day)**						
<1	29	4 (13.8%)	25 (86.2%)	38	17 (44.7%)	21 (55.3%)
1–2	31	7 (22.6%)	24 (77.4%)	27	10 (37.0%)	17 (63.0%)
3–4	44	11 (25.0%)	33 (75.0%)	44	13 (29.5%)	31 (70.5%)
5–9	120	12 (10.0%)	108 (90.0%)	103	33 (32.0%)	70 (68.0%)
10–19	362	35 (9.7%)	327 (90.3%)	357	94 (26.3%)	263 (73.7%)
≥20	747	71 (9.5%)	676 (90.5%)	352	73 (20.7%)	279 (79.3%)
p for trend		p < 0.006			p < 0.001	
**Education**						
Primary	214	7 (3.3%)	207 (96.7%)	118	9 (7.6%)	109 (92.4%)
Vocational	534	50 (9.4%)	484 (90.6%)	241	53 (22.0%)	188 78.0%)
Secondary	461	59 (12.8%)	402 (87.2%)	421	122 (29.0%)	299 (71.0%)
High	124	24 (19.4%)	100 (80.6%)	141	56 (39.7%)	85 (60.3%)
p for trend		p < 0.001			p < 0.001	
**Occupational classification**						
Non-economically active	322	22 (6.8%)	300 (93.2%)	359	68 (18.9%)	291 (81.1%)
Employed	883	112 (12.7%)	771 (87.3%)	506	160 (31.6%)	346 (68.4%)
Unemployed	128	6 (4.7%)	122 (95.3%)	56	12 (21.4%)	44 (78.6%)
p for trend		p > 0.05			p < 0.004	
**Place of residence**						
Rural	673	60 (8.9%)	613 (91.1%)	374	75 20.1%)	299 (79.9%)
Urban						
Up to 50 000	241	19 (7.9%)	222 (92.1%)	167	49 (29.3%)	118 (70.7%)
50 000–200 000	173	28 (16.2%)	145 (83.8%)	154	42 (27.3%)	112 (72.7%)
Over 200 000	246	33 (13.4%)	213 (86.6%)	226	74 (32.7%)	152 (67.3%)
p for trend		p < 0.009			p < 0.001	
**Monthly income**						
Less than 1000 PLN	376	20 (5.3%)	356 (94.7%)	304	55 (18.1%)	249 (81.9%)
From 1000 to 1500 PLN	309	27 (8.7%)	282 (91.3%)	253	69 (27.3%)	184 (72.7%)
From 1501 to 2000 PLN	293	43 (14.7%)	250 (85.3%)	158	51 (32.3%)	107 (67.7%)
From 2001 to 3000 PLN	201	25 (12.4%)	176 (87.6%)	127	46 (36.2%)	81 (63.8%)
Over 3000 PLN	153	26 (27.0%)	127 (83.0%)	78	19 (24.6%)	59 (75.6%)
p for trend		p < 0.001			p < 0.002	
**Awareness of smoking health consequences**						
Yes	1158	126 (10.9%)	1032 (89.1%)	845	225 (26.6%)	620 (73.4%)
No	175	14 (8.0%)	161 (92.0%)	76	15 (19.7%)	61 (80.3%)
p		p > 0.05			p > 0.05	
**Perceiving some kinds of cigarettes less harmful than others**						
May be less harmful	325	41 (12.6%)	284 (87.4%)	266	80 (30.1%)	186 (69.9%)
All are equally harmful	1008	99 (9.8%)	909 (90.2%)	655	160 (24.4%)	495 (75.6%)
p		p > 0.05			p > 0.05	
**Intention to quit**						
Intend to quit smoking within the next month	169	17 (10.1%)	152 (89.9%)	84	16 (19.0%)	68 (81.0%)
Consider quitting smoking within the next 12 months	303	43 (14.2%)	260 (85.8%)	267	65 (20.6%)	212 (79.4%)
Will quit smoking but not within the next 12 months	263	33 (12.5%)	230 (87.5%)	203	50 (24.6%)	153 (75.4%)
Do not intend to quit smoking	598	47 (7.9%)	551 (92.1%)	357	109 (30.5%)	248 (69.5%)
p for trend		p < 0.04			p < 0.02	

Among men, it was observed that the prevalence of use of flavored cigarettes decreased with age - from 14% at the age of 20-29 years to 4.2% over the age of 60 years (p for trend <0.04). Similarly, younger women aged 15-39 smoked flavored cigarettes more often than older women, and among women aged over 60 percentage of respondents smoking flavored decreased by half (15.2%; p for trend <0.001). The highest percentage of male and female flavoured cigarettes smokers was noted among those that have been smoking for 5-9 years. Among women, higher percentages of users of flavoured cigarettes were among those who smoked fewer cigarettes. Men who mostly smoked flavored cigarettes, consumed from 3 to 4 cigarettes per day (25.0%). The percentage of smokers of flavoured cigarettes increased with their level of education reaching 19.4% in men and close to 40% in women. More employed male and female smokers use cigarettes with menthol, vanilla, candy than regular cigarettes than among economically non-active or unemployed smokers. The highest prevalence of flavoured cigarettes use was among women from large cities (over 200 000 of inhabitants; p for trend <0.009) and men living in areas with a population of 50 000-200 000. Male smokers with highest income (over 3000 PLN) and female smokers earning from 2001 to 3000 PLN were the highest smokers of flavoured cigarettes. Prevalence of use of cigarettes with flavours was also higher in the group aware of smoking health consequences than those unaware. Similarly, a higher percentage of flavoured cigarettes was consumed among those who perceived that some kinds of cigarettes were less harmful than others than those who assumed that all are equally harmful. An interesting observation was that in women, the prevalence of use of flavored cigarettes increased with declining likelihood to quit (p for trend < 0.02). Over 30% of female smokers who do not intend to quit used flavoured cigarettes.

### Univariate analysis

Female smokers are more likely to smoke flavoured cigarettes (OR = 3.8; 95% CI: 2.4–3.8; p < 0.001). There was a higher likelihood of smoking flavoured cigarettes among female smokers based on age group. Smokers older than 20 years and less than 60 years of age have a higher likelihood of smoking flavoured cigarettes. Smokers between the ages 20–29 (OR = 2.9; 95% CI: 1.8–4.8; p < 0.001), ages 30–39 (OR = 2.8; 95% CI: 1.7–4.6; p < 0.001), ages 40–49 (OR = 2.3; 95% CI: 1.4–3.8; p < 0.001) and 50–59 (OR = 2.2; 95% CI: 1.3–3.6; p < 0.001) are more likely to smoke flavoured cigarettes relative to all other age groups, using smokers over age 60 as the reference group. Using 20 cigarettes per day as the reference point, smokers that smoke fewer than 20 cigarettes per day were more likely to smoke flavoured cigarettes. Smokers that smoke fewer than 5 cigarettes per day had the highest likelihood of smoking flavoured cigarettes (OR = 2.7; 95% CI: 1.9–3.8; p < 0.001), than smokers who smoke 5–9 cigarettes daily (OR = 1.7; 95% CI: 1.2–2.4), and smokers who smoke between 10–19 cigarettes per day (OR = 1.4; 95% CI: 1.12–1.9; p < 0.01) compared to subjects consuming twenty or more cigarettes daily.

Smokers with higher education have the highest probability of smoking flavoured cigarettes (OR = 8.5; 95% CI: 4.8–15.1; p < 0.001), followed by those in secondary school (OR = 5.1; 95% CI: 3.0–8.6; p < 0.001), and those in vocational schools (OR = 3.0; 95% CI: 1.7–5.2; p < 0.001). Smokers in primary school were the reference point. There was an increased probability of smoking flavoured cigarettes among people who are non–economically active (OR = 2.2; 95% CI: 1.3–3.7; p < 0.01), using unemployed smokers as a reference point. Smokers living in urban areas have a higher likelihood of smoking flavoured cigarettes relative to smokers living in rural areas (city 50 000–200 000 inhabitants vs. rural OR = 1.8; 95% CI: 1.3–2.5; p < 0.001; (city over 200 000 people vs. rural OR = 1.9; 95% CI: 1.5–2.6; p < 0.001). Smokers earning over 1500 PLN were over twice as likely to use flavoured cigarettes relative to those earning less than 1000 PLN (Table 2).

**Table 2:** Odds ratios (OR) and 95% confidence intervals (CI) for use of flavoured cigarettes to selected socio-demographic characteristics – Global Adult Tobacco Survey Poland (2009–2010)

**Characteristic**	**Flavoured cigarettes users**			**Univariate logistic regression**		**Multivariate logistic regression**^**a**^	
	**n**	**%**	**95% CI**	**OR**	**95% CI**	**OR**	**95% CI**
**Gender**							
Male	140	10.5	8.9–12.1	1.00	Reference	1.00	Reference
Female	240	26.1	23.3–28.9	3.00***	2.39–3.79	2.28***	1.62–3.23
**Age (years)**							
15–19	7	12.3	3.8–20.8	1.59	0.64–3.94	3.91	0.73–20.92
20–29	85	20.5	16.6–24.4	2.92***	1.76–4.85	2.73*	1.14–6.53
30–39	96	19.7	16.2–23.2	2.78***	1.68–4.57	1.49	0.67–3.35
40–49	89	16.9	13.7–20.1	2.30***	1.39–3.80	1.35	0.62–2.97
50–59	82	16.1	12.9–19.3	2.18***	1.32–3.62	1.36	0.64–2.90
> 60	21	8.1	4.8–11.4	1.00	Reference	1	Reference
**Number of years of smoking (years)**							
<5	13	10.7	5.2–16.2	0.69	0.37–1.28	0.41	0.16–1.07
5–9	54	21.8	16.7–26.9	1.40	0.98–1.99	0.68	0.36–1.30
≥10	312	16.5	14.8–18.2	1.00	Reference	1.00	Reference
Daily cigarette consumption (cigarettes per day)							
<5	62	29.1	23.0–35.2	2.72***	1.93–3.84	2.77	0.97–7.91
5–9	45	20.2	14.9–25.5	1.68**	1.16–2.43	1.40	0.83–2.37
10–19	129	17.9	15.1–20.7	1.45**	1.12–1.88	1.02	0.71–1.46
≥20	144	13.1	11.1–15.1	1.00	Reference	1.00	Reference
**Education**							
Primary	16	4.8	2.5–7.1	1.00	Reference	1.00	Reference
Vocational	103	13.3	10.9–15.7	3.03***	1.75–5.22	1.87	0.95–3.67
Secondary	181	20.5	17.8–23.2	5.10***	3.01–8.65	2.33*	1.17–4.64
High	80	30.2	24.7–35.7	8.54***	4.84–15.06	3.19**	1.44–7.06
**Occupational classification**							
Employed	272	19.6	17.5–21.7	1.40	0.82–2.40	2.55	0.93–6.98
Non–economically active	90	13.2	10.7–15.7	2.25**	1.35–3.73	3.27*	1.17–9.13
Unemployed	18	9.8	5.5–14.1	1.00	Reference	1.00	Reference
**Place of residence**							
Rural	135	12.9	10.9–14.9	1.00	Reference	1.00	Reference
Urban							
Up to 50 000	68	16.7	13.1–20.3	1.35	0.98–1.85	0.98	0.61–1.58
50 000–200 000	70	21.4	17.0–25.8	1.84***	1.34–2.53	1.64*	1.04–2.59
Over 200 000	107	22.7	18.9–26.5	1.98***	1.49–2.62	1.65*	1.08–2.51
**Monthly income**							
Less than 1000 PLN	75	11.0	8.6–13.4	1.00	Reference	1.00	Reference
From 1000 to 1500 PLN	96	17.1	14.0–20.2	1.66**	1.14–2.42	1.03	0.57–1.85
From 1501 to 2000 PLN	94	20.8	17.1–24.2	2.17***	1.48–3.17	1.22	0.65–2.26
From 2001 to 3000 PLN	71	21.6	17.1–26.1	2.21***	1.47–3.33	1.02	0.52–2.01
Over 3000 PLN	45	19.5	14.4–24.6	2.02**	1.27–3.20	0.97	0.45–2.09
**Awareness of smoking health consequences**							
Yes	351	17.0	15.8–19.2	1.62*	1.07–2.47	1.38*	1.07–2.15
No	29	11.6	7.6–15.6	1.00	Reference	1.00	Reference
**Perceiving some kinds of cigarettes less harmful than others**							
May be less harmful	121	20.5	17.2–23.8	1.40	0.99–2.00	1.40**	1.08–1.80
All are equally harmful	259	15.6	13.9–17.3	1.00	Reference	1.00	Reference

^a^Fully adjusted model including all analysed characteristics.

^*^p ≤ 0.05.

^**^p ≤ 0.01.

^***^p ≤ 0.001.

It was also observed that being aware of smoking health consequences increased one’s likelihood of smoking flavoured cigarettes relative to those who are unaware (OR = 1.6; 95% CI: 1.1–2.5; p < 0.05). There was no significant association of likelihood between smoking aromatized cigarettes and monthly incomes. There was no significant association of smoking flavoured cigarettes based on the number of years one has been smoking and number of cigarettes smoking per day.

### Multivariate analysis

Results from the multivariate analysis are displayed in Table 2. Most of the results in the univariate analysis were similar to these results in terms of socio-demographic characteristics, with very minimal differences. Similar to the univariate analysis, female smokers still prefer smoking flavoured cigarettes than male smokers (OR = 2.3; 95% CI: 1.6-3.2; p < 0.001). Smokers between the ages 30-59 had no significant associations with smoking aromatized cigarettes, but smokers between the ages 20-29 have a higher likelihood of smoking flavoured cigarettes using smokers over age 60 as a reference point (OR = 2.7; 95% CI: 1.1-6.5; p < 0.05).

There was no significant association between number of years smoked, number of cigarettes smoked per day and smoking flavoured cigarettes. Results showed that smokers in high and secondary schools were less than half as likely to smoke flavoured cigarettes, compared with results from the univariate analysis (OR = 3.2; 95% CI: 1.4-7.1; p < 0.01: OR = 2.3; 95% CI: 1.2-4.6; p < 0.05 respectively) relative to smokers with primary education. Non-economically active smokers have a higher likelihood to smoke cigarettes with exotic flavours such as menthol or candy, relative to the results in the univariate section (OR = 3.3; 95% CI: 1.2-9.1; p < 0.05).

Results also showed that a smoker’s monthly income had no significant association with smoking aromatized cigarettes, as was also observed in the univariate analysis. A difference in the multivariate analysis is that people who believe that cigarettes vary in the level of harm are more likely to smoke flavoured cigarettes (OR = 1.4; 95% CI: 1.1-1.8; p < 0.01) compared to those who assume all cigarettes have the same level of harm.

## Discussion

Consistent with other epidemiology studies till date on flavoured cigarettes use, GATS displayed that female smokers tend to prefer flavoured cigarettes compared to male smokers [8,22,23]. The scientific literature is consistent with this finding for adult smokers [8,24]. Unfortunately, we do not have any potential observation or previous data on flavoured cigarettes use in Poland to determine whether this proportion has changed or consistently remains the same. GATS also revealed that younger smokers until age 29 years use cigarettes with characterizing flavour far more likely than older smokers [25-27]. The lack of significant association with the age group 15-19 years could be explained by the small cell sizes for this youngest group of flavoured cigarettes smokers, but the positive direction of association remained. The proportion of menthol smokers among all cigarette smokers has been shown to be higher among adolescents than among adults in most, racial or ethnic groups with the exception of African American smokers [8].

The reasons for preferring flavoured cigarettes including menthol cigarettes among younger, novice smokers are many including tobacco industry aggressive advertisement or promotion tactics addressed to specific social and demographic groups, especially young people and women that may alter the attractiveness of the product [28]. Packaging – colors, branding, shape of packs or additional descriptions (“delicious”, “super-slims”, “velvet mint”, “frissons”) also play an important role in creating false beliefs that these products are less harmful than others, less harassing and more pleasant in general [29-31]. Removing brand descriptors from packs significantly reduces measures of appeal and taste, particularly for brands with flavour descriptors, such as cherry and vanilla. Plain packs are significantly less likely to be associated with positive images, such as glamour, sophistication, and slimness [30].

On the other hand, some additives like menthol enhance the taste, reduce the harshness, and stimulate cold receptors, providing a sensation of coolness. Smokers find them tastier as well as easier to inhale especially for new smokers [32]. Study by Hersey et al. based on data from the National Youth Tobacco Survey indicated that menthol cigarettes are a starter product that may be associated with smoking uptake by youth [27]. Moreover menthol cigarettes seem to serve as a conditioned stimulus that reinforces the rewarding effects of smoking [33].

In GATS another important factor increasing use of cigarettes with aromatic additive is having a higher level of education. Factors that may potentially contribute to more common use of such products between respondents with higher levels of education may include general increased awareness on health, attitudes and social norms. People with higher levels of education may consider or be more aware of health risks of smoking and choose products advertised as less harmful or products that claim to increase mental or physical performance, unless based on misleading perceptions. In accordance with these outcomes, we noted that people aware of negative health consequences of smoking cigarettes are more likely to use flavoured brands. Similarly, there was an association between perceiving less harmful cigarettes with aromatized cigarettes. Several previous studies conducted with other populations have demonstrated comparable results [34].

In GATS we noted similar rates of respondents perceived some kinds of cigarettes less harmful than others (26.2%) compared to the results of the International Tobacco Control (ITC), where overall; 27.8% of current smokers believed that some cigarettes could be less harmful; with 22.4% of current smokers in the UK, and 24.3% in France endorsing beliefs that some brands might be less harmful [34]. This proportion was higher only in Germany (36.7%). Compared to German smokers, Poles were less likely to hold beliefs that some brands might be less harmful. It should be stressed that across the countries, among those who believed that some cigarettes were less harmful than others; close to 50% thought taste was an indicator of harm [34]. In addition, the most popular reasons participants gave for selecting the brands they usually smoked were taste and satisfaction which reached 72% among smokers. Despite this, over a fifth (21.8%) of participants overall gave health as reasons for selecting their brands which also contribute to justifying our results [34].

Living in a large city was another factor associated with use of flavoured cigarettes. Hypothetically it can be related to greater rates of people with higher education in large cities compared to rural settings. Another reason could be marketing strategies and more intensive targeting of this subgroup of population relative to the rural population. Unfortunately we lack data on tobacco industry campaigns. Nevertheless, due to a somewhat restrictive ban of tobacco advertisement, promotions including those in all mass media tobacco industries have to use more sophisticated, direct attitudes. It is also easier and more efficient to approach higher numbers of people in large cities, such as during mass events or in bars or discos. Another issue is lack of ban of displaying tobacco products at points of sale. Inhabitants of big cities due to more developed infrastructure and greater concentration of points of sales are more exposed on this type of marketing. On the other hand, the sociocultural aspect of menthol cigarette smoking among rural and urban respondents is a probable fundamental reason in explaining this issue.

Our findings which show higher odds of using flavoured cigarettes among economically non active respondents is probably related to the characteristic of this group covering mostly young people; for example young women occupied with home and child care, pupils or students who as we previously indicated are more prone to use of flavoured cigarettes.

GATS data analysis revealed a lack of association between income and the use of flavoured cigarettes. A review of the evidence on this topic by Caraballo et al. showed that data on income and the use of menthol cigarettes is almost nonexistent in the literature [8]. Figures from the US National Survey on Drug Use and Health (NSDUH) showed that adult smokers with family incomes of less than $50 000 were more likely to smoke menthol cigarettes than adult smokers with higher family incomes. Nonetheless, there is also no previous available statistics from Poland for comparison.

There was also no association between duration of smoking and number of cigarettes smoked per day with use of aromatized cigarettes. It might be expected that those who smoke less cigarettes and beginner smokers smoking for short period would prefer flavoured cigarettes and then due to rising dependency for example switch to traditional cigarettes. Although we found higher percentage of non-flavoured cigarettes users among female consuming twenty cigarettes per day or more this association was not confirmed in the multivariate analysis for entire population. In other studies results are inconsistent regarding the frequency and direction of switching between menthol and nonmenthol cigarettes [10]. No studies were identified by Rising et al. in their review that directly addressed whether current smokers started smoking with menthol cigarettes and then switched to non-menthol cigarettes [10].

But it also may suggest that smokers at different stages or trajectories of cigarette smoking are susceptible to tobacco advertisement and promotion of flavoured brands regardless on the length of habit and the level of dependency.

As has been concluded based on analysis of documents derived from tobacco companies menthol is a prominent design feature used by cigarette manufacturers to attract and retain new smokers [13,35,36]. Marketing studies showed that the companies carefully research the menthol segment of the market in order to recruit smokers to their brands. The industry tracks menthol cigarette usage by age, gender and race to inform product development and marketing decisions. Furthermore, the tobacco industry knows consumers perceive menthol as healthier than non-menthol cigarettes, and this was the intent behind marketing [13,28,35].

However there are some promising observations from the study that explored how menthol smokers might react if menthol cigarettes were banned. O’Connor et al. noted that over 60% of menthol smokers in the United States might respond to a ban on menthol cigarettes reducing or quitting smoking (36.5% of current menthol cigarettes smokers would try to stop smoking, 27.1% would smoke less than now if such cigarettes were banned) [37]. Finally 14.7% declared that would switch to another brand, 10.6% would add menthol on their own [37]. There is no such studies regarding flavoured cigarettes use by Poles but increasing the information on determinants of smoking of flavoured cigarettes is fundamental for developing and implementing more effective tobacco control measures and enact legislation at the national and European levels.

### Study limitations

GATS providing nationally representative figures on status of different tobacco products use based on a high number of respondents covering all 16 voivodeships of our country [17]. However this study is not free of limitations [38]. There are well-known potential limitations resulting from use of self-reports and cross-sectional design, but these issues were discussed in previous papers and should not significantly decrease the quality of the study [18,19,21]. It should be also mentioned that in relation to current analysis information on smoking initiation with particular type of cigarettes (regular or aromatized) and switching or not to different types of tobacco were missing. Although GATS questionnaire included questions on beliefs about harmfulness of regular and different cigarettes, important information about reasons for choosing brands, taste preferences or beliefs about indicators of less harmful cigarettes were missing. This missing information makes it unable for us to compare GATS data with other results and should be a subject of further, in-depth investigations.

## Conclusions

It is well known that use of tobacco products would not continue without the initiation and maintaining of their use by youth and adults [10]. So far, data on smoking flavoured cigarettes and its social associates in Poland is undeveloped. Overall, there is a paucity of data on this topic. There is the need to improve Polish national surveillance system for accurate measurement and monitoring of tobacco epidemic including use of products with flavoured additives as well as tracking tobacco industry activities and new marketing strategies. To our knowledge, GATS is the first study assessing simultaneous impact of a number of socio-demographic variables on use of cigarettes with characterizing flavour among Poles. In spite of some limitations of GATS we have indicated several significant associations of use of flavoured cigarettes among adults in our country. Figures make it evident that flavoured including menthol cigarettes are disproportionately used by particular groups of young adults under age 30 and women in Poland.

These findings should encourage policy makers to undertake further steps to curb tobacco epidemic. Regulations on additives and forms of pack branding remain a potential tool for decreasing the appeal of tobacco products to women and young people. Apart from ban of flavoured additives and slim brands, restricting potentially misleading information from cigarette packages and plain packaging should be enforced and implemented. This is essential to protect younger age groups and women in particular. Moreover, interventions to raise awareness on smoking health risks and counter-advertising to reduce misperceptions about the lower risk of flavoured or slim cigarette brands are necessary. This study clearly shows that other aspects of tobacco control in Poland should be expanded to increase the comprehensiveness of anti-tobacco policies in order to decrease the smoking rates.

Enactment and enforcement of amendments of European Union’s Tobacco Products Directive considered among other bans on menthol cigarettes sale would lead to a substantial progress in tobacco control in Poland and other EU countries.

## Competing interests

The authors declare that they have no conflicts of interests.

## Authors’ contributions

DK as a representative of the World Health Organization (CO POL) coordinated GATS in Poland, outlined the paper, discussed core ideas, and prepared the final manuscript. BU co-drafted the paper. AST prepared the dataset, did the data analysis. TMD discussed core ideas and commented extensively on drafts. All authors read and approved the final manuscript.

## Pre-publication history

The pre-publication history for this paper can be accessed here:

http://www.biomedcentral.com/1471-2458/14/127/prepub
